# A Genome-Wide Association Study in Psoriasis Patients Reveals Variants Associated with Response to Treatment with Interleukin-17A Pathway Inhibitors

**DOI:** 10.3390/genes16101187

**Published:** 2025-10-13

**Authors:** Dimitra Ioakeimidou, Efterpi Zafiriou, Themistoklis Giannoulis, Olga Kouvarou, Kalliopi Gerogianni, Dimitrios P. Bogdanos, Theologia Sarafidou, Kalliopi Liadaki

**Affiliations:** 1 Department of Biochemistry and Biotechnology, School of Health Sciences, University of Thessaly, 41500 Larissa, Greece; dioakeimidou@uth.gr (D.I.); sarafid@bio.uth.gr (T.S.); 2 Department of Dermatology, Faculty of Medicine, School of Health Sciences, University of Thessaly, 41500 Larissa, Greece; zafevi@med.uth.gr (E.Z.); kouvarouolga@gmail.com (O.K.); kalgerog@yahoo.com (K.G.); 3 Department of Animal Science, University of Thessaly, Gaiopolis, 41334 Larissa, Greece; themisgia@gmail.com; 4 Department of Rheumatology and Clinical Immunology, Faculty of Medicine, School of Health Sciences, University of Thessaly, Biopolis, 41500 Larissa, Greece; bogdanos@uth.gr

**Keywords:** psoriasis, Interleukin 17 inhibitors, Interleukin 17 receptor inhibitor, genome-wide association study

## Abstract

Background/Objectives: Psoriasis is currently treated with biologics targeting the IL-17A signaling, which plays a major role in immune response and keratinocyte hyperproliferation. These include inhibitors of IL-17A and/or its heterodimer with IL-17F (Secukinumab, Ixekinumab and Bimekizumab) and the receptor IL17-RA (Brodalumab). Although these drugs are safe and highly effective, there is significant variability in response among patients. This can be partly attributed to the patients’ genetic background, thus pointing to the need to identify pharmacogenetic markers for treatment response. Methods: The study involved 88 Greek patients who were treated with inhibitors of the IL-17A signaling for at least 6 months. Patients were classified as responders and non-responders according to the change in Psoriasis Area Severity Index. A total of 730,000 variants were genotyped and analyzed for association with the 3-month and 6-month responses to treatment. Results: The analysis identified 21 variants which were associated with the response, showing statistical significance after Bonferroni correction. These include variants located in protein coding genes (*TP63*, *NRG1*, *SCN8A*, *TAF9*, *TMEM9*, *SMIM36*, *SYT14*, *BPIFC*, *SEZ6L2*, *PCARE*), as well as intergenic and long non-coding RNA intronic variants. The functional significance of the variants was assessed using in silico analysis and for several variants, a link with immune processes was proposed. Notably, *rs11649499* status, which was associated with complete clinical remission at 3 months, may influence key lipid mediators involved in psoriasis. Conclusions: This GWAS identified novel variants that could be utilized upon validation in larger populations as predictive markers regarding patient response to drugs targeting the IL-17A pathway.

## 1. Introduction

Psoriasis is an autoimmune disease with genetic and environmental etiology [1]. The treatment of moderate to severe psoriasis includes conventional systemic therapies, which act as broad immunosuppressants, and the new era biologic agents, which block specific cytokines involved in the disease process. The latter include tumor necrosis factor-α (TNF-α), Interleukin (IL)-23 and IL-17 inhibitors [2].

The IL-17 signaling pathway is targeted by four drugs that have been approved for adults with moderate to severe psoriasis: Secukinumab (Cosentyx^®^) in 2015, Ixekizumab (Taltz^®^) in 2016, Brodalumab (Siliq^®^ in the USA, kyntheum^®^ in EU) in 2017 and Bimekizumab (Bimzelx^®^) in 2023. In 2020, Ixekizumab was approved for the treatment of pediatric psoriasis (ages 6 to under 18). Apart from Brodalumab, all other drugs have also been approved for the treatment of psoriatic arthritis and ankylosing spondylitis. In particular, Secukinumab inhibits IL-17A [3], Ixekinumab inhibits IL-17A, alone or in complex with IL-17F [4], Bimekizumab is a dual IL-17A and 17F inhibitor [5], and Brodalumab is an IL-17RA antagonist [6]. IL-17A and IL-17F are members of the IL-17 family, which share the highest homology at the protein level (50%), and the respective genes are located on chromosome 6 within a 46 Kb distance. IL-17A and IL-17F, both as homodimers and as heterodimers, bind to the same receptor complex, comprising IL-17RA and IL-17RC subunits [7]. Upon binding to the receptor, a cytoplasmic protein (Act1) is recruited at the IL-17RA subunit and operates as a docking station for different TRAF proteins, resulting in the activation of several independent signaling pathways, such as Nuclear Factor kappa B (NF-κB) and Mitogen-Activated Protein kinase (MAPK), which cause de novo inflammatory gene transcription [8]. In psoriatic skin, T helper (Th)17 cells secrete high levels of IL-17A and IL-17F, which act on immune and non-immune cell types, such as keratinocytes [9]. Once activated, keratinocytes exhibit abnormal proliferation and differentiation and produce inflammatory mediators and chemokines, amplifying the inflammatory response [10].

The advantages of IL-17 signaling inhibitors include their good tolerance together with high levels of effectiveness, with more than 80% of the patients achieving 75% improvement on the Psoriasis Area and Severity Index (PASI75) and more than 68% reaching PASI90, from a 3-month treatment which persists up to a year (reviewed in [11]). The results of the clinical trials have also been validated in real-world evidence settings [12,13,14,15]. In addition, recent meta analyses have demonstrated that all IL-17 signaling inhibitors, together with the IL-23 inhibitors, have the fastest clinical response, compared to other biologic and systemic treatments [16], and have high survival rates, with data not yet reported about the most recently approved Bimekizumab [17].

Despite the fact that these drugs are ranked among the most successful psoriasis treatments, there is a significant proportion of patients that do not experience clinical remission. As genetic polymorphisms are important factors in individual variation in drug responses, pharmacogenomics pro-vides insight into predicting individual treatment responses. Previous association studies have reported pharmacogenetic markers associated with response to various biologic treatments in psoriasis (reviewed in [18]). In the case of IL-17 inhibitors, there have been a few reports investigating the association of single-nucleotide polymorphisms (SNPs) with treatment response based on candidate gene approaches. Specifically, *HLA-Cw6*, the primary psoriasis genetic susceptibility allele [19,20] and five variants in the protein-coding and untranslated regions of the *IL-17A* gene [21] have been tested for association with response to Secukinumab and Ixekinumab, with no positive results. In addition, the investigation of 21 pre-selected SNPs related to psoriasis and immunological diseases identified five SNPs associated with response to Secukinumab and Ixekinumab, after 3 and 12 months of therapy [22]. Finally, the analysis of 417 SNPs, chosen based on their implication in immune response and inflammatory pathways, identified 13 SNPs which, in combination with the *HLA-Cw6* status of the patients, were associated with response to Secukinumab in different treatment periods [23].

To our knowledge, a non-hypothesis-driven genetic association study for inhibitors of IL-17 signaling, which could identify variants associated with treatment response on a genome-wide significance level, is missing. This was the main objective of the present study, in which we have performed a pharmacogenomic genome-wide association study (GWAS) of treatment response to all the inhibitors of the IL-17A, signaling in a cohort of 88 Greek patients with moderate to severe psoriasis. Our secondary objective was the functional investigation of the associated variants exploring in silico analyses.

## 2. Materials and Methods

### 2.1. Patient Selection and Classification

This study involved 88 patients, who were recruited from the outpatient clinic of the Dermatology Department of the University General Hospital in Larissa, Greece, during the period between 2020 and 2024. All patients were diagnosed with moderate-to-severe psoriasis, according to their baseline PASI score that was equal or higher than 5, a threshold which is consistent with many real-world studies [14,15,24]. In addition, all patients were older than 18 years of age and had undergone treatment with biologic therapies targeting the IL-17A pathway for at least 6 months, a time period during which their clinical data were recorded. Patients received monotherapy treatment according to the recommended dosing schemes. Secukinumab was administered subcutaneously, at a dose of 300 mg at weeks 0, 1, 2, 3 and 4, followed by a maintenance dose of 300 mg every 4 weeks. Ixekizumab was given with an initial dose of 160 mg, followed by an 80 mg dose at weeks 2, 4, 6, 8, 10, 12, and subsequent injections every 4 weeks. Bimekizumab was administered at 320 mg (given as two subcutaneous injections of 160 mg each) at weeks 0, 4, 8, 12 and 16, followed by injections every 8 weeks. Brodalumab was administered with a 210 mg subcutaneous injection at weeks 0, 1 and 2, followed by injections every 2 weeks. There were 11 patients who were treated with more than one of the aforementioned medications at different time periods. These patients were included in the study once, because they exhibited similar response to treatment to the different drugs. Twenty out of the 88 patients had been previously treated with apremilast, and this cohort subset has been included in our previous pharmacogenetic analysis of treatment response to apremilast [25]. The previous use of biologic agents was recorded for all patients. The response to treatment was evaluated based on the reduction in the PASI score at baseline and following 3 and 6 months of treatment, with a time window of two weeks. Specifically, patients with PASI50 (50% reduction in the PASI score) were considered as non-responders, while patients reaching PASI75 and higher were classified as responders. While PASI75 is considered a satisfactory therapeutic goal and an efficacy criterion for psoriasis treatment, the introduction of new biologic agents suggests that PASI90 or higher may be considered as the optimal therapeutic standard in psoriasis [26,27]. In this respect, the patients were also classified as responders when they achieved PASI90 and PASI100, and as non-responders when they exhibited less than 75% reduction in the PASI score. The comparison of biologic-naïve status between responder and non-responder patients was performed using Fisher’s statistical significance exact test [28]. In addition, the comparison of weight and BMI status between responder and non-responder patients was performed using the nonparametric Kruskal–Wallis test [29], as the data deviated from the normal distribution. All analyses were performed using the SPSS program, version 20. The study was conducted according to the World Medical Association Declaration of Helsinki—Ethical Principles for Medical Research Involving Human Participants and was approved by the Research Ethics Committee of the University General Hospital of Larissa, University of Thessaly (ECA #19/14-11-2019 and ECA 11/02/08-02-2022). All patients who participated in the study provided written informed consent. No personal information was provided about the participants during the study.

### 2.2. Genotyping and Quality Control Procedure

Genomic DNA was extracted from the patient’s peripheral blood (200 or 400 μL) using the PureLink Genomic DNA kit (Thermo Fisher Scientific, Waltham, MA, USA) according to the manufacturer’s instructions. DNA integrity was assessed by agarose gel electrophoresis and DNA concentration and purity were determined using a Quawell spectrophotometer (San Jose, CA, USA). Typical DNA yields obtained were 100–500 ng/μL with 260/280 absorbance ratios between 1.8–2 and 200 ng of genomic DNA (at a concentration of 50 ng/μL) were shipped in dry ice to the Human Genomics Facility (HuGe-F) of Erasmus Medical Centre in The Netherlands. Genotyping was performed using the Illumina Infinium Global Screening Array Multiple Disease v3.0 (Illumina, Eindhoven, The Netherlands), which contains approximately 730,000 variants, and the results were obtained in .ped and .map files.

The quality control of the genotyping data included the following steps: (i) SNP-level missingness threshold set at 0.01, thus excluding SNPs not present in >1% of all the individuals, (ii) individual-level missingness threshold set at 0.01, thus removing individuals with >1% missing genotype data, (iii) application of a high heterozygosity filter in order to remove individuals who deviate ±5 SD from the samples’ mean heterozygosity rate, (iv) exclusion of SNPs with minor allele frequency (MAF) ≤ 3%, (v) pruning of the data based on Linkage Disequilibrium (LD) values, with the threshold set at r^2^ > 0.8 (pruning was based on pairwise correlation with the window size for the calculation set to 50 SNPs and the window increment shifting 5 SNPs forward), (vi) exclude individuals based on relatedness, which was defined through identity-by-descent (IBD) analysis with a threshold set at a PI_HAT value > 0.0625. All the above analyses were performed using the PLINK software (PLINK 1.9) [30].

### 2.3. Association Analysis

All variants that remained after filtering were tested for association with response to treatment implementing allelic, dominant, recessive and genotypic models using the PLINK software. The raw *p* values were adjusted based on Bonferroni correction for multiple testing and the adjusted *p* value of <0.05 was used as the threshold for statistical significance. The odds ratio (OR) was calculated with 95% Confidence Interval (CI) using non-responders as the case group.

The chromosomal location, the minor allele frequencies and the genomic coordinates of the variants were retrieved from the Ensembl (https://www.ensembl.org/index.html) [31] and the dbSNP (www.ncbi.nlm.nih.gov/snp/) databases [32] (both accessed on 20 August 2025). As the genotypic data obtained from HuGe-F included genomic coordinates using the human genome GRCh37 assembly, the Ensembl database was used to convert those coordinates according to the human genome GRCh38 assembly.

### 2.4. In Silico Analysis

The bioinformatics source HaploReg (v4.2) (https://pubs.broadinstitute.org/mammals/haploreg/haploreg.php) [33] (accessed on 5 September 2025) was used to analyze the localization of SNPs in regions of DNase hypersensitivity, and regions of regulatory motifs as well as histone modifications marking promoters and enhancers. In addition, Genotype–Tissue Expression (GTEx) portal (www.gtexportal.org) (accessed on 1 September 2025) was used to evaluate the expression quantitative trait locus (eQTL) effect of the variants in tissues related to psoriasis, such as skin and immune cells, using the threshold of *p* value < 0.05.

## 3. Results

### 3.1. Patients and Treatment Response

Initially, the study included 91 patients, who were all genotyped for 725,497 variants. Following the genotyping data quality control, one patient was excluded due to missing genotype data, and two patients were further excluded due to cryptic relatedness (they appear to have more than fourth-degree relation, as their PI_HAT values were >0.0625). Finally, the remaining 88 patients passed the extreme heterozygosity filters. Regarding SNPs, 36,411 were removed due to SNP missingness, then a subsequent filter excluded 296,236 due to MAF ≤ 3% and pruning for LD excluded additional 95,584 SNPs. Thus, 297,266 SNPs were finally employed for the association analysis regarding response to IL-17 signaling inhibitors.

Table 1 summarizes the physical and clinical characteristics of the studied population. Among the 88 patients, 63 were males and 25 were females. Their mean baseline weight was 91.8 kg, and their mean baseline body mass index (BMI) was 30.1. The mean age of disease onset was reported at 30.7 years and the mean age of treatment with the drugs was 45.5 years, with a mean baseline PASI score of 14.9. In addition, 35.2% of the patients were diagnosed with psoriatic arthritis and 59.1% had nail psoriasis. The most common comorbidities reported in 48.9% of the patients were cardiovascular disease, hypertension, dyslipidemia and diabetes mellitus. Regarding the treatment history of the patients, 54.5% of the subjects were naïve for biologic agents, with half of them having received only local therapy and the other half had received single or combined treatment with conventional or systemic drugs (e.g., methotrexate, cyclosporin, acitretin and apremilast).

Regarding treatment, 62 patients received Secukinumab, 11 Ixekizumab, 7 Bimekizumab and 17 patients received Brodalumab. The response to treatment was evaluated 3 months (early response) and 6 months after the beginning of the treatment. Specifically, at 3 months, 65 out of 76 patients (85%) reached PASI75, 52 out of 80 patients (65%) reached PASI90 and 31 out of 59 patients (52%) reached PASI100, indicating complete clearance of clinical signs. At 6 months of treatment, 67 out of 73 patients (91%) achieved PASI75, 65 out of 78 patients (83%) had PASI90 and 43 out of 56 patients (76%) were super-responders (PASI100). The denominations differ between 3 and 6 months, because seven patients missed their scheduled appointment by more than two weeks, which was the time window that was used for the estimation of response, so their PASI scores were not included in the study. The observed response rates are comparable to the results obtained from clinical trials [11]. Biologic naïvety has been reported as a factor related to patient response to biologic therapy [34]. The comparison of biologic-naïve status between responder and non-responder groups of patients revealed that lack of previous biologic treatment was a significant contributor (*p* = 0.004) with regard to an early (3 month) PASI100 response, in agreement with previous findings [35]. Since many patients are overweight or obese, we investigated whether weight or BMI (Table 1) is correlated with response efficacy. We observed no significant differences in these parameters between responder and non-responder subgroups (*p* values were between 0.298 and 0.61).

### 3.2. Identification of SNPs Associated with Response to Treatment with Inhibitors of IL-17A Signaling

The association analysis of 297,266 SNPs, based on the allelic model, identified 21 SNPs that exhibited statistical significance after Bonferroni adjustment (Table 2). Furthermore, association analysis was performed for the group of patients (*n* = 76) that received drugs targeting only IL-17A and IL-17F, but not their receptor (Supplementary Table S1). This analysis identified 15 SNPs, eight of which were also found when all the inhibitors of the IL-17A signaling pathway were considered (Table 2).

All but one of the associated SNPs had minor allele frequencies significantly higher in the non-responder groups, therefore constituting risk alleles. Only *rs11649499* is protective, as the frequency of the minor allele (C) was significantly higher in the super-responder (PASI100) group following 3 months of treatment. For the same period, 3 SNPs (*rs9848736*, *rs4252217* and *rs12448418*) were associated with treatment response using PASI75 as threshold. Regarding the 6-month treatment, 16 SNPs (Table 2) were associated with achievement of PASI75 and 2 SNPs (*rs75504215* and *rs10166913*) were associated with achievement of PASI90. Notably, *rs10166913* was detected in both PASI75 and PASI90 groups. No associations were found for the super-responder cohort following 6 months of treatment and for the group of patients that reached PASI90 after 3 months.

We calculated the MAFs of all the SNPs associated with treatment response and compared them with the frequencies reported in the Ensembl database. The MAFs ranged from 3.2% to 12%, with the exception of *rs11649499* (MAF 35%) and are close to the MAFs reported for European populations. As shown in Table 2, the odds ratios (ORs) for these associations are notably high compared to those typically observed in GWAS, suggesting strong effects; however, the low allele counts likely contribute substantially to these elevated values. Of note, the associations remained statistically significant after Bonferroni correction with 95% confidence intervals (CIs) that do not include 1.

The associated SNPs map on 13 different chromosomes. Based on their genomic location they are divided into the following categories: (i) SNPs that are missense variants: *rs11649499* in *SEZ6L2* and *rs10166913* in *PCARE*, (ii) SNPs that are intron or 5′/3′ untranslated (UTR) variants of protein coding genes: *rs6468095* and *rs17624997* in *NRG1*; *rs62279932* in *TP63*; *rs115692430* in *SCN8A*; *rs34437895* in *SYT14*; *rs77691176* in *BPIFC*; *rs115790464* in *TMEM9*; *rs9914970* in *SMIM36*; *rs75264797* in *KIF5C*; *rs4252217* in *AK6*, *RAD17* and *TAF9*; *rs41291977* 5′ prime UTR variant for *FKBP11* and 3′ prime UTR variant for *ARF3*, iii) SNPs that are intron variants for long non-coding RNAs (lncRNAs): *rs75504215* for *ENSG00000305441*; *rs78216879* for *LINC02006*; *rs2051337* for *LINC00907* and *rs7867365* for *ENSG00000289185*, and iv) SNPs that are intergenic (*rs9848736*, *rs12448418*, *rs74894123* and *rs17056507*).

### 3.3. In Silico Analysis of the Regulatory Potential of the SNPs Associated with Response to IL-17A Inhibitors

The regulatory potential of the identified variants in tissues relevant to psoriasis (skin and peripheral blood subpopulations) was examined using HaploRegv4.2., and the results are summarized in Table 3. This database reports the effect of the minor allele variants in altering the binding motifs for specific transcription factors (TFs), thus reflecting putative changes in gene expression. The identification of more than one binding motif for several variants (Table 3) is due to the fact that often the sequences of these binding motifs are overlapping. HaploReg also reports the effect of the minor allele variants in altering chromatin structure and generating DNase hypersensitivity sites (HSSs), which might result in increased expression of nearby genes. Finally, this database catalogs the effect of the variants in promoting histone modifications, by generating methylation and acetylation sites in promoters and enhancers, possibly affecting the expression of genes even at long range distances. Such modifications include monomethylation of the 4th lysine residue on histone H3 protein (H3K4me1), trimethylation of the 4th lysine residue on histone H3 (H3K4me3), acetylation of the 9th lysine residue on histone H3 (H3K9ac) and acetylation of the 27th lysine residue on histone H3 (H3K27ac).

We searched for genes whose expression correlates with the variants associated with response in tissues relevant to psoriasis and the results are summarized in Table 4. *rs11649499* is an eQTL for *PARG1* (located ~76 Kb upstream) in blood, for *CDIPTOSP* (located ~29 Kb upstream) in skin and for *GDPD3* Antisense RNA (located ~214 Kb downstream) in lymphocytes. In addition, *rs4252217* correlates with the expression of *CCDC125* (located ~37 Kb upstream) and *TAF9* in blood, *NAIPP2* (located ~723 Kb downstream) in skin, and correlates with changes in the splicing ratios of *CDK7* alternative transcripts (sQTL) in cultured fibroblasts. *CDK* is located 92 Kb upstream of *rs4252217* and encodes for cyclin dependent kinase 7. Table 4 also includes information about the functional role of the genes listed as eQTLs for the associated variants, and if available their putative involvement in the disease. The potential mechanisms by which these genes may influence treatment response remain largely speculative, and will be further explored in the discussion section, or unknown.

### 3.4. Replication Analysis

To date, a few studies have reported genetic associations with response to Secukinumab and Ikekinumab treatment [22,23]. In an effort to independently replicate previous findings, we investigated the association of the identified SNPs in our dataset. SNPs in *TLR2*, *TNF*, *SLC12A8*, *ZNF816A* and *TNFAIP3* genes were associated with 3-month response (measured as PASI90 and absolute PASI < 1) to Secukinumab and Ixekinumab in 19 psoriasis patients [22]. All the SNPs were included in our genotyping dataset, but *rs610604* was excluded during the filtering procedure. For the remaining SNPs (*rs11938228*, *rs1800629*, *rs651630* and *rs9304742*), we found no association with response (raw *p* values: *p* > 0.31, *p* > 0.16, *p* > 0.19, and *p* > 0.08, respectively). In addition, a previous study identified 13 variants associated with response to Secukinumab in 62 patients from Italy [23]. 5 out of the 13 variants were included in our genotyping dataset, i.e., 3 *HLA-Cw6* variants (*rs12191877*, *rs4406273* and *rs10484554*) and 2 *LTA* variants (*rs1800683* and *rs909253*). Of those, two variants (*rs4406273* and *rs1800683*) remained after the filtering procedure. We replicated the association of *rs4406273* before Bonferroni adjustment (raw *p* value = 0.01).

## 4. Discussion

The present study is the first pharmacogenomic analysis of response to IL-17A, IL-17F and IL-17RA inhibitors using a genome-wide association approach. We identified 21 SNPs, which are associated with response in patients with moderate to severe psoriasis for both 3 months and 6 months after treatment. Ideally, from a clinical standpoint, discovering genetic markers of prediction of the patient’s remission of clinical signs early in the treatment is of ultimate importance.

Our analysis revealed that *rs11649499* minor allele C is associated with patient super-response after 3 months of treatment. *rs11649499* (221 G > C) determines a missense substitution in the SEZ6L2 protein (Arg74>Pro), which has an established role in neuronal development. Although this variant is not an eQTL for *SEZ6L2*, its minor allele correlates with differential expression of the nearby genes *PARG1*, *GDPD3 antisense RNA* and *CDIPTOSP.* PARG1, apart from other functions, is part of a complex with histone H3K4 methyltransferases MLL3/MLL4, suggesting a role in epigenetic gene regulation of target genes located at long range distances. *GDPD3 antisense RNA*, located ~214 Kb downstream, targets *GDPD3* encoding the glycerophosphodiesterase 7 isoform, which acts on lysophospholipids to produce lysophosphatidic acid (LPA) [36]. There are many reports regarding the role of LPA in the pathogenesis of psoriasis. An increase in serum LPA concentrations in patients with psoriasis and in skin psoriatic lesions in psoriasis-like mouse models have been demonstrated [37]. LPA induced the activation of its receptor on keratinocytes [37] and macrophages [38], triggering inflammasome activation during psoriasis development, while the inhibition of LPA receptors alleviated skin symptoms in psoriasis-like mouse models and decreased keratinocyte proliferation in the lesion [39]. Furthermore, *CDIPTOSP* is a transcribed pseudogene, located ~250 bp upstream of the 3′ of its relevant protein coding gene *CDIPT*, which in turn encodes for the protein that catalyzes phosphatidylinositol biosynthesis. It is well-known that pseudogenes have been implicated in the regulation of parental genes, through competitive binding of common miRNAs [40]. A recent study demonstrated that treatment with biologics, specifically Ixekinumab, could affect glycerophospholipid metabolism and facilitate the transition of metabolic status from a pro-inflammatory to an anti-inflammatory phenotype in patients with psoriasis [41]. The identification of a variant associated with patient super-response appears to influence key lipid mediators involved in psoriasis emphasizes the significance of lipidomics for the identification of biomarkers relevant to the diagnosis and treatment of psoriasis, as recently suggested [42].

GWAS have consistently identified variants that map predominantly to non-coding regions of the genome, where functional characterization remains limited. However, speculations about the putative function of the variants can be obtained from data of the ENCODE project, which provides a huge amount of information regarding functional elements in the human genome, as well as from the Genotype–Tissue expression project, which among others provides information about the correlation between genetic variants and changes in gene expression in various human cell lines and tissues. In this context, *rs4252217* and *rs9914970*, associated with the achievement of PASI75 after 3 months and 6 months, respectively, have been correlated with differential gene expression, constituting QTLs. *rs4252217* is an eQTL for *TAF9*, in which it is located, as well as for *CCDC125* and *NAIPP2 (NAIP pseudogene 2)* and an sQTL for *CDK7.* TAF9 (TATA-box binding protein-associated factor 9) is one of the TAFs associated with transcription factor II D complex that plays a major role in the initiation of RNA polymerase II-dependent transcription. *CCDC125* variants have been associated in GWAS with lymphocyte and neutrophil counts (https://www.ebi.ac.uk/gwas/genes/CCDC125) (accessed on 10 September 2025). NAIP, encoded by the relevant functional gene, is a constituent of the inflammasome and a regulator of innate immune signaling [43]. *CDK7* has been implicated in inflammation, based on a recent study which demonstrated that disrupting the RNA polymerase II transcription cycle through CDK7 pharmacological inhibition ameliorates inflammatory arthritis [44]. Along the same lines, *rs9914970*, an intronic variant in *SMIM36*, has been identified as eQTL for the nearby (~34 Kb) *MMD* gene in whole blood (HaploRegv4.2) [45]. *MMD* encodes for monocyte to macrophage differentiation factor, which regulates ERK and Akt activation and TNF-α production in macrophages [46]. Taken all together, our findings imply that both variants are correlated with expression of genes involved in inflammatory processes.

A direct link to psoriasis can be suggested about variant *rs62279932* associated with patient response after 6 months of treatment. It maps within *TP63* encoding Tumor Protein P63, which plays a crucial role in regulating the proliferation and differentiation of keratinocytes [47]. A role for TP63 in the development or maintenance of psoriasis is supported by the fact that ΤP63 expression appears to be involved in epidermal remodeling, even in the early stages of the disease [48] and TP63 major isoform is differentially regulated in psoriasis [49]. Furthermore, a previous study showed that TP63 activation (induced by ozone), promotes keratin 10 expression and accelerates basal keratinocyte differentiation, resulting in improvement in psoriatic lesions [50]. *rs62279932* alters binding motif for TFs involved in immune response (Smad3, AP4) and is located within DNase HSS in keratinocytes and histone modification marks in promoters and enhancers in keratinocytes (H3K4me1, H3K27ac, H3K4me3) and T CD8+ cells (H3K4me3). Based on the above, *rs62279932* can presumably affect *TP63* expression.

A bidirectional communication between neurons and immune cells in the development of immune-mediated disorders, including psoriasis, has been previously proposed [51]. In line with that, our analysis identified variants located within genes with established roles in neuronal development, i.e., *NRG1* (*rs6468095* and *rs17624997*, which are not in high LD) and *SCN8A* (*rs115692430*). Regarding *NRG1*, in vivo and in vitro evidence support its involvement in the immune system dysregulation [52]. NRG1 can reduce levels of pro-inflammatory cytokines through NF-kB signaling [53] and exhibits anti-inflammatory effects in cultured keratinocytes and in atopic dermatitis-like mice [54]. Recently, NRG1 was identified as a characteristic biomarker for hypertrophic scar, a prevalent chronic inflammatory disorder [55], in which IL-17 expression is markedly increased [56]. Although there is no information available from the eQTL analysis, these variants are located within DNase HSS and enhancer modification marks (H3K4me1, H3K27ac) in keratinocytes and fibroblasts, presumably affecting *NRG1* expression. *SCN8A* encodes for the sodium voltage-gated channel alpha subunit 8, which, apart from neuronal cells, is also expressed in keratinocytes and macrophages [57,58]. Heterozygous knockout mice for *SCN8A* exhibit reduced expression of IL-6 and IFN-γ, increased IL-10 levels and reduced inflammatory responses to lipopolysaccharide challenge [59]. According to GTEX, *rs115692430* correlates with *SCN8A* expression; however, it does not in tissues relevant to psoriasis. It has been proposed that a cross talk between nociceptive neurons and the local immune system, specifically Th17 cells appears to result in a vicious circle that supports classical symptoms of psoriasis, such as pruritus, pain and hyperalgesia [60]. In this aspect, it will be interesting to further explore the participation of these variants in psoriasis pathogenesis.

Finally, variants *rs10166913*, *rs115790464*, *rs41291977* and *rs77691176* have indications for potential regulatory roles, relevant to autoimmune and/or skin disorders. Variant *rs10166913* (1739C>T) determines a missense substitution in the PCARE protein (Thr580Met), which so far is involved in photoreceptor function. Τhe in silico analysis shows that *rs10166913* is associated with enhancer modifications (H3K4me1, H3K27ac) in skin cells, which could potentially affect expression of the nearby ubiquitously expressed gene *CLIP4* (~25 Kb downstream), previously identified as self-antigen in systemic lupus erythematosus (SLE) associated with disease onset and activity [61]. *rs41291977* maps in the 5′ UTR region of *FKBP11* mRNA and affects histone modification at enhancers and promoters in skin and blood cells. FKBP11 (FK506 binding protein 11) possesses peptidyl-prolyl cis-trans isomerase activity and binds immunosuppressive drugs [62]. Highly expressed FKBP11 is involved in the pathogenesis of auto-inflammatory diseases including SLE and Crohn’s disease [63,64]. *rs115790464* is located within *TMEM9*, encoding transmembrane protein 9, which has been implicated in various cellular mechanisms, including inflammation [65]. The variant is associated with histone modification marks in promoters and enhancers in skin cells, which could affect *TMEM9* expression. In lung adenocarcinoma, TMEM9 activates the MEK/ERK/STAT3 pathway and induces vascular endothelial growth factor (VEGF) expression [66], a pathway also involved in psoriasis. Another variant, *rs77691176*, lies within *BPIFC*, which has been detected as a causative gene for autosomal dominant trichilemmal cysts, a condition characterized by scalp cysts derived from hair follicles and consisting of keratinized epidermal wall surrounding hair keratin [67]. In addition, *BPIFC* was involved in the infiltration of naïve T CD4^+^ cells and T CD8^+^ cells in hypertrophic cardiomyopathy [68]. In silico analysis showed that *rs77691176* appears to be associated with histone modifications in promoter in Th-naïve and mononuclear cells, possibly affecting *BPIFC* expression in the respective cells.

To our knowledge, this is the first GWAS which investigates all inhibitors targeting the IL-17A signaling. We identified a highly promising variant (*rs11649499)* associated with early super-response to treatment, which appears to influence key lipid mediators involved with immune pathways and psoriasis. In addition, the in silico functional predictions for several variants showed associations with immune-related processes. However, for the majority of variants, the lack of supporting functional data limits direct interpretation, and their potential involvement in the drug’s mechanism of action remains speculative. It is also possible that the associated variants might be in high LD with other variants that play a direct role in the drug’s mechanism of action. This is a limitation of the study, as is often the case with many variants identified through GWAS, which typically lack a direct functional role in the underlying pathophysiology of a phenotype. Treatment response is a multifactorial trait, influenced by both genetic and environmental factors, such as anthropometric characteristics, clinical factors and lifestyle. The potential impact of many environmental factors which cannot be quantified in a clinical setting cannot be accounted for and this constitutes another limitation of the study. However, we have shown that weight and BMI status of the patients does not correlate with the response outcome. Finally, the main limitation of this study is the small size of the population studied, which requires validation in independent studies and larger cohorts. Upon validation, the identified variants—particularly the one associated with early super-response—hold potential for clinical application in guiding personalized therapy approaches.

## Figures and Tables

**Table 1:** Clinical and demographic characteristics of patients.

Patient Characteristic	Value
Gender (males/females)	63/25
Age of disease onset (years, mean ± SD)	30.7 (± 13.2)
Age of treatment onset (years, mean ± SD)	49.5 (± 13.2)
Baseline PASI (mean ± SD)	14.9 (± 8.2)
Baseline weight (kg, mean ± SD)	91.8 (± 16.7)
Baseline BMI (kg/m^2^, mean ± SD)	30.1 (± 5.3)
Patients with comorbidities, *n* (%)	43 (48.9)
Patients with nail psoriasis, *n* (%)	52 (59.1)
Patients with psoriatic arthritis, *n* (%)	31 (35.2)
Biologic-naïve patients, *n* (%)	48 (54.5)

Note: SD—standard deviation; BMI—body mass index; PASI—psoriasis area severity index.

**Table 2:** Statistically significant SNPs associated with the response to treatment with inhibitors of IL-17A/F and IL-17RA.

Treatment Response	SNP	Location (bp)	F_R	F_NR	*p* Raw	*p* Adj	OR	95% CI
3 m: R (*n* = 65) > PASI75; NR (*n* = 11) < PASI50	*rs9848736 (C/A)*	Chr. 3: 190.810.697	0.061	0.454	1.32 × 10^−7^	0.0392	12.71	4.2–38.3
	*rs4252217 (C/T)*	Chr. 5: 69.369.635	0	0.227	3.25 × 10^−8^	0.0096	N/A	N/A
	*rs12448418 (C/T)*	Chr. 16: 57.504.782	0.007	0.273	4.14 × 10^−8^	0.0123	48.38	5.4–427.8
3 m: R (*n* = 31) = PASI100; NR (*n* = 28) < PASI75	*rs11649499 (G/C)*	Chr. 16: 298.971.112	0.693	0.196	6.21 × 10^−8^	0.0184	0.108	0.05–0.25
6 m: R (*n* = 67) > PASI75; NR (*n* = 6) < PASI50	*rs6468095 (C/T)*	Chr. 8: 32.306.960	0.082	0.666	8.11 × 10^−9^	0.0024	22.36	5.8–86.2
	*rs9914970 (C/T)*	Chr. 17: 55.456.223	0.074	0.583	1.41 × 10^−7^	0.0421	17.36	4.6–64.7
	*rs17056507 (T/C)*	Chr. 13: 59.392.598	0.044	0.5	3.79 × 10^−8^	0.0112	21.33	5.3–86.3
	*rs115692430 (G/A)*	Chr. 12: 51.609.277	0.052	0.5	1.81 × 10^−7^	0.0539	18.14	4.6–70.9
	*rs78216879 (A/G)*	Chr. 3: 153.571.831	0.022	0.416	8.95 × 10^−9^	0.0026	31.19	6.2–157.3
	*rs62279932 (T/C)*	Chr. 3: 189.766.500	0.029	0.416	9.42 × 10^−8^	0.028	23.21	5.1–106.1
	*rs34437895 (C/A)*	Chr. 1: 209.938.683	0.007	0.25	8.19 × 10^−7^	0.0167	44.32	4.2–470.3
	*rs115790464 (C/T)*	Chr. 1: 201.144.928	0.022	0.333	1.37 × 10^−6^	0.0252	21.83	4.2–114.6
	*rs74894123 (G/A)*	Chr. 2: 36.293.569	0.022	0.333	1.37 × 10^−6^	0.0252	21.83	4.2–114.6
	*rs75264797 (A/G)*	Chr. 2: 148.972.862	0.022	0.333	1.37 × 10^−6^	0.0252	21.83	4.2–114.6
	*rs17624997 (G/A)*	Chr. 8: 32.280.133	0.067	0.5	2.23 × 10^−6^	0.0307	13.89	3.7–51.9
	*rs7867365 (C/T)*	Chr. 9: 13.772.938	0.0074	0.25	8.19 × 10^−7^	0.0167	44.32	4.2–470.3
	*rs41291977 (T/G)*	Chr. 12: 48.937.896	0.015	0.333	1.02 × 10^−7^	0.0303	33	5.3–208.1
	*rs77691176 (G/A)*	Chr. 22: 32.421.411	0.015	0.333	1.02 × 10^−7^	0.0303	33	5.3–208.1
	*rs2051337 (A/G)*	Chr. 18: 42.624.131	0.015	0.333	1.02 × 10^−7^	0.0303	33	5.3–208.1
	*rs10166913 (G/A)* *	Chr. 2: 29.072.523	0	0.333	1.23 × 10^−11^	3.65 × 10^−6^	N/A	N/A
6 m: R (*n* = 65) > PASI90; NR (*n* = 13) < PASI75			0	0.231	2.23 × 10^−8^	0.0069	N/A	N/A
	*rs75504215 (T/C)*	Chr. 15: 40.910.502	0	0.231	2.23 × 10^−8^	0.0069	N/A	N/A

Note: 3 m—3 months; 6 m—6 months; R—responders; NR—non-responders; F_R—frequency of the alternative allele in responders; F_NR—frequency of the alternative allele in non-responders; *p* adj.—*p* adjusted; OR—odds ratio, CI—Confidence Interval; PASI—Psoriasis Area Severity Index. N/A: Not Applicable. SNP location is based on GRCh38 assembly (Ensembl). The parenthesis of each SNP includes the alternative first and the reference allele second. The asterisk indicates that this SNP was detected using different PASI thresholds.

**Table 3:** In silico analysis of the regulatory potential of the SNPs associated with response to inhibitors of IL-17A and IL-17RA.

SNP	Binding Motifs Altered	DNase HSS	Enhancer/Promoter Histone Modifications
*rs11649499*	ZBTB7A		
*rs10166913*	CTCF, Ncx		fibroblasts, melanocytes
*rs12448418*	Hdx		fibroblasts
*rs9848736*	Pou2f2		
*rs17056507*	Stat, Smad3, Smad4	B, T cells	B, Th memory, Th17, Th-naïve, Treg, T CD8+-naïve, T CD8+ memory cells
*rs74894123*	ERalpha-a, Egr1, Ets, Sin3Ak20, YY1		
*rs75504215*	Egr1	fibroblasts, melanocytes	keratinocytes, fibroblasts, melanocytes; monocytes, Th-naïve, Th, Treg cells
*rs78216879*	Foxl1		melanocytes
*rs2051337*			
*rs7867365*			
*rs4252217*	BCL, BHLHE40, E2F, ELF1, HEN1, HEY1, Rad21, Sin3Ak20, YY1	keratinocytes, fibroblasts, melanocytes; T, B cells	keratinocytes, fibroblasts, melanocytes; Th, Th17, Treg cells
*rs6468095*	Hlx1	keratinocytes	keratinocytes, fibroblasts
*rs17624997*	Foxa, Foxl1, Myf4, Zfp105	keratinocytes, fibroblasts	keratinocytes, fibroblasts
*rs62279932*	Ets, Myc, Smad3, AP4	keratinocytes	keratinocytes; T CD8+-naïve cells
*rs115692430*	CEBPG, DMRT2, Myb4, Sox-2, -7, -10, -13, -14, -16, -18, -19, YY1	T cells	Th, T CD8+-naïve cells
*rs9914970*	STAT, Irf, HDAC2, TCF12, RXRA, TATA		fibroblasts; Th-naïve cells
*rs77691176*	Pou2f2, Pou3f3, p300		Th-naïve, mononuclear cells
*rs115790464*	Nanog, Sox17		fibroblasts, keratinocytes
*rs34437895*	ELF1, Egr1, Evi1, HDAC2, Pou2f2, TATA, p300	fibroblasts	fibroblasts, melanocytes, keratinocytes; T, B, Th-naïve, Th, Th17 cells
*rs75264797*	LRH1		melanocytes
*rs41291977*			fibroblasts, melanocytes; monocytes, B, Th-naïve, Th memory, Th17 cells

Note: Th—T helper; Treg—T regulatory; HSS—hypersensitivity sites.

**Table 4:** eQTL analysis of the variants associated with response to inhibitors of IL-17A signaling.

SNP	Gene Symbol	P	Tissue	Functional Role
*rs11649499*	*PAGR1*	3.50 × 10^−5^	Whole Blood	In complex with histone H3K4 methyltransferases PAGR1 regulates gene expression epigenetically
	*ENSG00000275371* *(GDPD3 antisense RNA)*	1.60 × 10^−4^	Lymphocytes	GDPD3, encoded by the target gene, produces lysophosphatidic acid, which has a known role in psoriasis pathogenesis
	*CDIPTOSP* *(CDIPT opposite strand, pseudogene)*	1.60 × 10^−5^	Skin	CDIPT, encoded by the relevant functional gene, catalyzes phosphatidylinositol biosynthesis
*rs4252217*	*CCDC125*	2.00 × 10^−5^	Whole Blood	*CCDC125* variants were associated with lymphocyte and neutrophil counts
	*TAF9*	2.20 × 10^−5^	Lymphocytes	TAF9 is involved in the initiation of RNA polymerase II-dependent transcription
	*NAIPP2* *(NAIP pseudogene 2)*	1.10 × 10^−3^	Skin	NAIP, encoded by the relevant functional gene, is a constituent of the inflammasome and a regulator of innate immune signaling
	*CDK7*	*CCDC125*	Fibroblasts	CDK7 is implicated in inflammation

Note: *PAGR1*—PAXIP1-associated glutamate rich protein 1; *GDPD3*—glycerophosphodiester phosphodiesterase domain containing 3; *CDIPT*—CDP-diacylglycerol-inositol 3-phosphatidyltransferase; *CCDC125*—Coiled-coil domain containing 125; *TAF9*—TATA-box binding protein-associated factor 9; *NAIP*—NLR family apoptosis inhibitory protein; *CDK7*—cyclin-dependent kinase 7.

## Data Availability

The original contributions presented in the study are included in the article/Supplementary Materials, further inquiries can be directed to the corresponding author.

## Supplementary Materials

The following supporting information can be downloaded at: https://www.mdpi.com/article/10.3390/genes16101187/s1, Table S1: SNPs associated with response to treatment with IL-17A and IL-17F inhibitors. Table S1: SNPs associated with response to treatment with IL-17A and IL-17F inhibitors. Table S2: Association analysis for 3-month treatment response setting PASI75 as the threshold. Table S3: Association analysis for 3-month treatment response setting PASI90 as the threshold. Table S4: Association analysis for 3-month treatment response setting PASI100 as the threshold. Table S5: Association analysis for 6-month treatment response setting PASI75 as the threshold. Table S6: Association analysis for 6-month treatment response setting PASI90 as the threshold. Table S7: Association analysis for 6-month treatment response setting PASI100 as the threshold.

## Abbreviations

The following abbreviations are used in this manuscript:

IL	Interleukin
PASI	Psoriasis Area and Severity Index
GWAS	Genome-wide association study
eQTL	Expression quantitative trait locus
SNP	Single-nucleotide polymorphism
