# Supplementary material for: A Genome-Wide Association Study in Psoriasis Patients Reveals Variants Associated with Response to Treatment with Interleukin-17A Pathway Inhibitors
Source: Genes (Basel). 2025 Oct 13;16(10):1187. doi: 10.3390/genes16101187 (PMC12564490; doi:10.3390/genes16101187)
Supplement: Supplementary file 1 [file genes-16-01187-s001.zip › Table S1. Statistically significant SNPs associated with the response to treatment with inhibitors of IL-17A and IL-17F.pdf]

Supplementary Table S1. Statistically significant SNPs associated with the response to treatment with inhibitors of IL-17A and IL-17F.

**Treatment response**	**SNP**	**Location (bp)**	**F_R**	**F_NR**	**p raw**	**p adj**	**OR**	**95% CI**
3 m: R (n=55) >PASI75; NR (n=10) <PASI50	*rs9848736 (C/A)*	Chr. 3: 190.810.697	0.054	0.5	2.43E-08	0.0072	17.34	5.2 - 57.7
	rs58258089 (T/G)	Chr. 1: 7.199.082	0	0.25	8.90E-08	0.0264	N/A	N/A
3 m: R (n=28) =PASI100; NR (n=25) <PASI75	*rs11649499 (G/C)*	Chr. 16: 298.971.112	0.679	0.16	7.55E-08	0.0224	0.09	0.04 - 0.23
6 m: R (n=58) >PASI90; NR (n=12) <PASI75	*rs75504215 (T/C)*	Chr. 15: 40.910.502	0	0.25	3.70E-08	0.011	N/A	N/A
6 m: R (n=60) >PASI75; NR (n=6) <PASI50	*rs78216879 (A/G)*	Chr. 3: 153.571.831	0.008	0.416	9.50E-11	2.82E-05	8.5	8.7 - 829.6
	rs10166913 (G/A)	Chr. 2: 29.072.523	0	0.333	1.34E-10	3.98E-05	N/A	N/A
	rs62279932 (T/C)	Chr. 3: 189.766.500	0.016	0.416	3.73E-09	0.0011	42.14	6.9 - 257.1
	rs6468095 (C/T)	Chr. 8: 32.306.960	0.075	0.667	5.41E-08	0.0016	24.67	6.2 - 97.6
	rs13321831 (G/A)	Chr. 3: 189.786.702	0.025	0.416	5.91E-08	0.0175	27.85	5.5 - 141.1
	rs9914970 (C/T)	Chr. 17: 55.456.223	0.067	0.583	7.57E-08	0.0225	19.6	5.1 - 75.8
	rs4471745 (G/A)	Chr. 17: 55.491.523	0.008	0.333	1.88E-08	0.0055	59.48	5.9 - 596.6
	rs75218991 (A/G)	Chr. 4: 83.866.410	0	0.25	3.02E-08	0.0089	N/A	N/A
	rs72743502 (A/G)	Chr. 5: 29.277.411	0	0.25	3.02E-08	0.0089	N/A	N/A
	rs2241223 (G/A)	Chr. 22: 21.054.902	0	0.25	3.02E-08	0.0089	N/A	N/A
	rs11016893 (C/T)	Chr. 10: 129.725.352	0.008	0.333	1.88E-08	0.0055	59.48	5.9 - 596.6

Note: 3 m.: 3 months; 6 m.: 6 months; R: responders; NR: non-responders; F_R: frequency of the alternative allele in responders; F_NR: frequency of the alternative allele in non-responders; p adj.: p adjusted; OR: odds ratio, PASI: Psoriasis Area Severity Index. Location of the SNPs is based on GRCh38 assembly (Ensembl). The parenthesis of each SNP includes first the alternative and second the reference allele
